# A systematic review and meta-analysis comparing the impact of tenofovir and entecavir on the prognosis of hepatitis B virus-related hepatocellular carcinoma patients undergoing liver resection

**DOI:** 10.3389/fphar.2024.1443551

**Published:** 2024-07-29

**Authors:** Lingbo Hu, Chao Yang, Yingli Qiao, Aidong Wang

**Affiliations:** ^1^ Department of Hepatopancreatobiliary Surgery, Taizhou Hospital of Zhejiang Province Affiliated to Wenzhou Medical University, Taizhou, Zhejiang, China; ^2^ Department of Hepatopancreatobiliary Surgery, Enze Hospital, Taizhou Enze Medical Center (Group), Taizhou, Zhejiang, China; ^3^ Department of Blood Purification, Taizhou Hospital of Zhejiang Province Affiliated to Wenzhou Medical University, Taizhou, Zhejiang, China

**Keywords:** hepatocellular carcinoma, entecavir, tenofovir, liver resection, meta-analysis, prognosis

## Abstract

**Background:**

Tenofovir (TDF) and entecavir (ETV) are highly effective and well-tolerated nucleos(t)ide analogs commonly prescribed for hepatitis B virus (HBV) treatment. Yet, it is unclear whether survival outcomes differ for HBV-related hepatocellular carcinoma (HCC) patients treated with ETV and TDF. Thus, this meta-analysis aimed to compare the prognostic effectiveness of ETV and TDF in HBV-related HCC patients.

**Methods:**

We comprehensively searched four databases, PubMed, Web of Science, Embase, and the Cochrane Library, to identify pertinent studies utilizing keywords “entecavir,” “tenofovir,” “hepatocellular carcinoma,” and “liver resection.” Our primary outcomes of interest encompassed overall survival (OS), recurrence-free survival (RFS), early recurrence, and late recurrence. The statistical effect size for these measures was expressed in terms of hazard ratios (HRs).

**Results:**

Our search yielded 10 studies encompassing 11 datasets involving 7,400 patients. Our meta-analysis revealed that patients treated with TDF achieved better OS (HR = 0.53; 95% confidence interval [CI] = 0.40–0.70, *p* < 0.0001), RFS (HR = 0.68; 95% CI = 0.57–0.80; *p* < 0.0001), early recurrence (HR = 0.80; 95% CI = 0.67–0.94; *p* < 0.0077), and late recurrence (HR = 0.64; 95% CI = 0.43–0.97; *p* = 0.0368). We detected publication bias potentially affecting OS but not RFS.

**Conclusion:**

Our findings demonstrated that TDF outperformed ETV regarding RFS for HBV-related HCC patients. However, to bolster the evidence and establish more conclusive conclusions, further validation via extensive and high-quality randomized controlled trials is essential.

**Systematic Review Registration:**

https://www.crd.york.ac.uk/prospero/#recordDetails, identifier CRD 42024542579.

## Introduction

Liver cancer is the sixth most common cancer globally and third leading cause of mortality (Sung et al., 2021), with hepatocellular carcinoma (HCC) constituting roughly 90% of the cases (Llovet et al., 2021). The considerable recurrence rate following liver resection contributes to an unfavorable prognosis in HCC patients (Llovet et al., 2021). Persistent hepatitis B virus (HBV) replication significantly elevates the risk of HCC recurrence. Nucleos(t)ide analog therapy, reducing the virus load, has the potential to substantially prolong overall survival (OS) and minimize tumor recurrence in HCC patients (Huang et al., 2015; Wang et al., 2020).

Tenofovir (TDF) and entecavir (ETV) are highly effective and well-tolerated nucleos(t)ide analogs used for HBV treatment. However, observations suggest that TDF may confer a significantly lower risk of HCC than ETV in patients with chronic hepatitis B (Choi et al., 2023). This finding prompts questions regarding whether the roles of TDF and ETV in the prognosis of HBV-associated HCC after liver resection differ. While some studies suggest a more favorable efficacy of TDF than ETV in the prognosis of HBV-related HCC patients (Choi et al., 2021; Qi et al., 2021), others have indicated similar efficacy of both drugs on the prognosis (Kao et al., 2023; Liang et al., 2024). Thus, we executed a meta-analysis to compare their prognostic efficacy following liver resection in HBV-related HCC patients.

## Materials and methods

This review has been registered in the PROSPERO database (registration No. CRD 42024542579).

### Search strategy

On 19 March 2024, we systematically searched the Web of Science, PubMed, Embase, and Cochrane Library utilizing a combination of MeSH terms and keywords, focusing on HCC, liver resection, entecavir, and tenofovir. Supplementary Table S1 lists the comprehensive details of the search strategy.

### Inclusion criteria

We used the PICOS criteria for inclusion, where P indicates that patients with HBV-related HCC received liver resection; I indicates that TDF was adopted after liver resection; C indicates that ETV was adopted after liver resection; O indicates that outcomes included overall survival (OS), recurrence-free survival (RFS), early recurrence, or late recurrence; and S indicates that retrospective studies and randomized controlled trials (RCTs) were legal.

### Exclusion criteria

Non-comparative studies, case reports, abstracts, comments, and reviews were excluded. In the cases of overlapping patient cohorts, only the foremost study, determined by factors such as superior quality, larger sample size, or the most recent publication, was included.

### Definition

OS and RFS were characterized as the duration from surgery to death and tumor recurrence, respectively. Early recurrence and late recurrence were specified as a recurrence within 2 years and 2 or more years post-liver resection, respectively.

### Quality assessment and data extraction

Two researchers independently performed quality assessment and data extraction using the Newcastle–Ottawa scale (NOS) for non-RCTs with scores of up to 9 points (Wells et al., 2014), and the Cochrane risk assessment tool was used for RCTs (Sterne et al., 2019). Study details, such as tumor characteristics, patient information, the first author, and the publication year, were extracted using pre-designed, standardized forms. Outcomes, including OS, RFS, early recurrence, and late recurrence, were extracted from original reports or via data conversion using ReviewManager software (version 5.3). Any disagreements between researchers were resolved by a third party.

### Statistical analysis

Hazard ratio (HR) and 95% confidence interval (CI) values were determined using the inverse variance method. Heterogeneity was evaluated through the Q statistic and I^2^, with I^2^ of 25% and 50% denoting low and moderate heterogeneity, respectively. Heterogeneity sources were explored using either meta-regression with the random-effects model for studies with I^2^ > 50% or subgroup analysis. A leave-one-out sensitivity analysis was applied to assess the robustness of the conclusion. Funnel plots were used to examine publication bias, and its influence on the results was further analyzed using the trim-and-fill method. All analyses were conducted using the R program (version 4.4.0). Statistically significant difference was deemed at *p* < 0.05.

## Results

### Study search and inclusion

**FIGURE 1:**
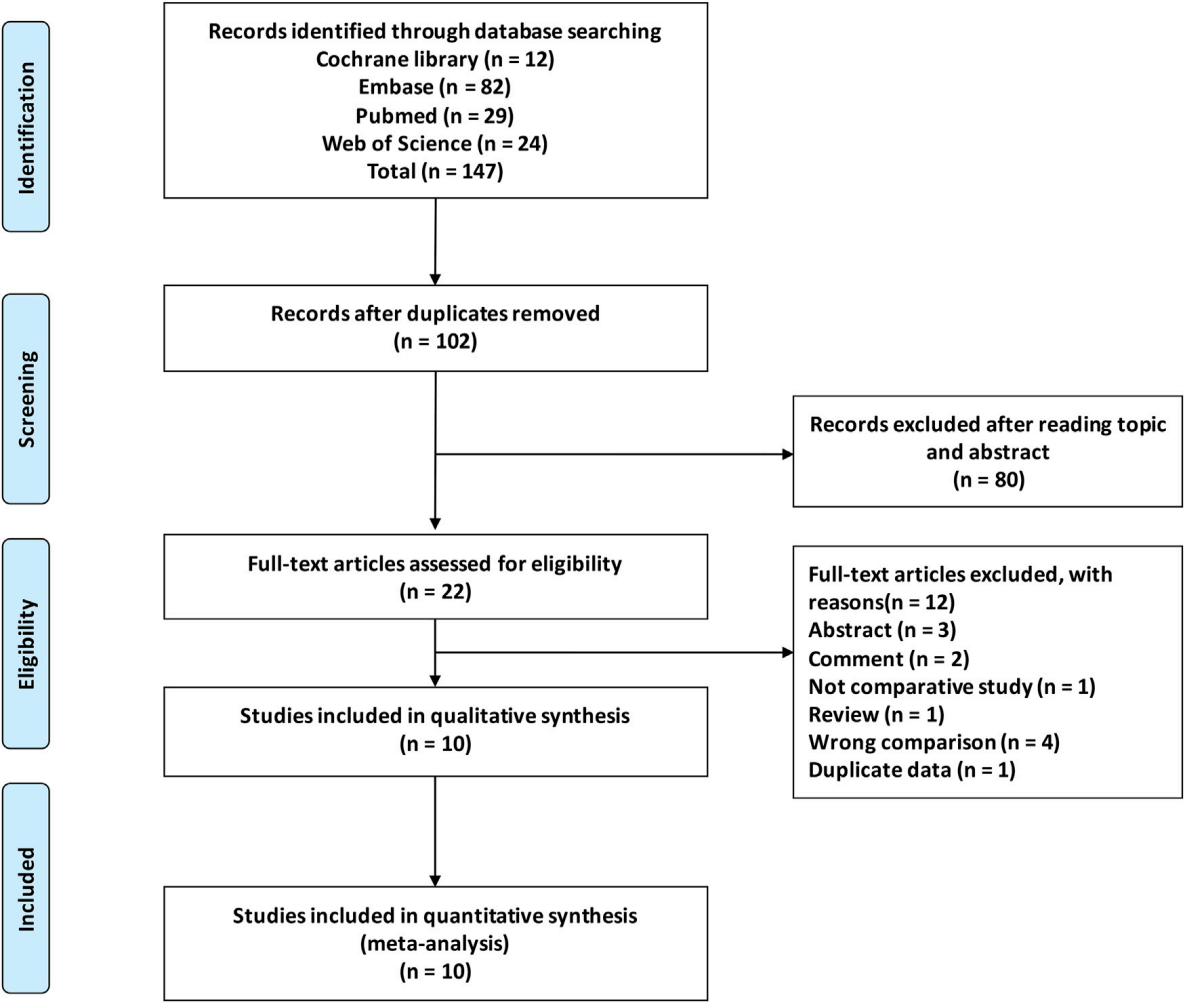
Flowchart depicting the selection process of studies.

A thorough search produced 147 articles, which were reduced to 102 after eliminating duplicates. After reviewing titles and abstracts, 22 records were retained. Twelve studies were excluded due to duplicated data, incorrect comparisons, and inappropriate article types (Figure 1). Consequently, this meta-analysis incorporated 10 studies (Zhang et al., 2018; Choi et al., 2021; Qi et al., 2021; Shen et al., 2022; Tsai et al., 2022; Wang et al., 2022; Kao et al., 2023; Li et al., 2023; Linye et al., 2023; Liang et al., 2024).

### Study characteristics

Ten studies, comprising 11 datasets and involving 7,400 patients, compared TDF and ETF efficacy in HBV-related HCC prognosis. Among them, nine were from China, and one was from Korea. Two were retrospective studies, one was an RCT, and the rest were retrospective studies using propensity analysis (including propensity-score matching, propensity-score overlap weighting, and inverse probability of treatment weighting). Three studies exclusively included patients at the Barcelona Clinical Liver Cancer Staging (BCLC) 0 or A stage. Table 1 lists the detailed patient and tumor characteristics.

**TABLE 1:** Features of the incorporated studies.

Study	NOS score	Design	Country	Group	Sample size	Age year	Gender male/female	HBV DNA copy/mL	HBeAg positive n (%)
Liang 2024	7	R	China	ETV	59	56 ± 12	53/6	12 (<1,000) 47 (≥1,000)	14 (23.7)
PO cohort				TDF	31	56 ± 14	28/3	12 (<1,000) 19 (≥1,000)	16 (51.6)
Liang 2024	7	R	China	ETV	51	59 ± 11	40/11	33 (<1,000) 18 (≥1,000)	17 (33.3)
PPO cohort				TDF	55	57 ± 12	45/10	34 (<1,000) 21 (≥1,000)	14 (25.5)
He 2023	NA	RCT	China	ETV	74	49.78 ± 11.95	66/8	57 (<2,000) 17 (≥2,000)	22 (29.7)
				TDF	74	50.97 ± 12.17	63/11	55 (<2,000) 19 (≥2,000)	16 (21.6)
Li 2023	9	PSM 1:1	China	ETV	989	58.3 ± 9.8	884/145	3.3 ± 1.8 (log copies/mL)	270 (27.3)
				TDF	989	58.4 ± 10.5	851/138	3.3 ± 1.8 (log copies/mL)	261 (26.4)
Kao 2023	9	PS overlap weighting	China	ETV	1,365	58.22 ± 11.14	1,143/222	NA	NA
				TDF	432	56.13 ± 10.79	367/65	NA	NA
Wang 2022	8	PSM 2:1	China	ETV	403	49.0 (18–80)^a^	344/59	116 (undetectable) 77 (<2,000) 210 (≥2,000)	117 (29.0)
				TDF	265	49.0 (18–79)^a^	231/34	73 (undetectable) 58 (<2,000) 134 (≥2,000)	77 (29.1)
Tsai 2022	8	PSM 2:1	China	ETV	146	56.4 ± 10.9	127/19	65 (undetectable) 15 (<2,000) 65 (≥2,000)	27 (18.5)
				TDF	73	56.5 ± 10.6	64/9	29 (undetectable) 11 (<2,000) 32 (≥2,000)	16 (21.7)
Shen 2022	9	IPTW	China	ETV	533	412 (≤60) 121 (>60)	450/83	162 (≤1,000) 371 (>1,000)	115 (21.6)
				TDF	62	52 (≤60) 10 (>60)	52/10	24 (≤1,000) 38 (>1,000)	15 (24.2)
Qi 2021	9	PSM 2:1	China	ETV	288	49.3 ± 10.6	248/40	131 (≤1,000) 157 (>1,000)	56 (23.0)
				TDF	144	49.9 ± 10.7	122/22	68 (≤1,000) 76 (>1,000)	29 (24.1)
Choi 2021	9	PSM 1:1	Korea	ETV	567	54.6 ± 8.6	430/137	238 (undetectable) 151 (<2,000) 178 (≥2,000)	137 (24.2)
				TDF	567	54.7 ± 9.3	433/134	236 (undetectable) 150 (<2,000) 181 (≥2,000)	149 (26.3)
Zhang 2018	7	R	China	ETV	126	55 (26–73)^a^	107/19	4.1 (3.0–5.1)^a^ (log copies/mL)	88 (69.84)
				TDF	107	52 (25–69)^a^	82/25	3.7 (3.0–4.7)^a^ (log copies/mL)	76 (71.03)

Cirrhosis n (%)	AFP ng/mL	BCLC stage	MVI positive n (%)	Tumor size cm	Tumor number single/multiple	Tumor differentiation	Satellite nodule n (%)
45 (76.3)	29 (<20) 30 (≥20)	29 (0/A) 30 (B)	30 (50.8)	6.2 ± 2.6	34/25	12 (P) 47 (M + H)	NA
20 (64.5)	17 (<20) 14 (≥20)	20 (0/A) 11 (B)	17 (54.8)	6.7 ± 2.6	22/9	10 (P) 21 (M + H)	NA
32 (62.7)	24 (<20) 27 (≥20)	29 (0/A) 22 (B)	17 (33.3)	6.4 ± 3.8	36/15	9 (P) 42 (M + H)	NA
37 (67.3)	26 (<20) 29 (≥20)	37 (0/A) 18 (B)	20 (36.4)	5.6 ± 3.2	37/18	15 (P) 40 (M + H)	NA
54 (73.0)	30 (<20) 44 (≥20)	21 (0) 53 (A)	17 (23.0)	3.05 ± 0.89	16/58	32 (P + M) 42 (H)	4 (5.4)
55 (74.3)	28 (<20) 46 (≥20)	32 (0) 42 (A)	19 (25.7)	2.91 ± 0.82	53/21	35 (P + M) 39 (H)	3 (4.1)
360 (36.4)	543.2 ± 6,339.4	98 (0) 747 (A) 144 (B)	477 (48.2)	4.2 (0.3–25.0)^a^	811/178	NA	NA
362 (36.6)	461 ± 3,065.8	120 (0) 743 (A) 126 (B)	471 (47.6)	4.1 (0.5–23.1)^a^	823/166	NA	NA
1,015 (74.36)	1,017 (<20) 230 (≥20)	155 (0) 806 (A) 404 (B)	NA	4.26 ± 3.24	NA	434 (P) 740 (M) 106 (H)	NA
334 (77.31)	313 (<20) 86 (≥20)	59 (0) 262 (A) 111 (B)	NA	3.78 ± 2.4	NA	114 (P) 259 (M) 34 (H)	NA
233 (57.8)	184 (<20) 219 (≥20)	29 (0) 330 (A) 44 (B)	94 (23.3)	5.5 (0.8–19.0)§	349/54	144 (P) 198 (M) 61 (H)	NA
164 (61.9)	128 (<20) 137 (≥20)	22 (0) 214 (A) 29 (B)	54 (20.4)	5.5 (0.9–19.5)§	229/36	96 (P) 130 (M) 39 (H)	NA
84 (57.5)	70 (<20) 76 (≥20)	34 (0) 112 (A)	50 (34.2)	2.6 ± 1.0	127/19	10 (P) 107 (M) 29 (H)	7 (4.8)
44 (60.3)	34 (<20) 39 (≥20)	19 (0) 54 (A)	25 (34.2)	2.7 ± 1.0	64/9	6 (P) 50 (M) 17 (H)	4 (5.5)
446 (83.7)	263(≤400) 270 (>400)	NA	205 (38.5)	8.5 (6.3–12.0)^b^	450/83	262 (P) 269 (M) 2 (H)	85 (15.9)
56 (90.3)	29 (≤400) 33 (>400)	NA	23 (37.1)	8.5 (6.5–11.0)^b^	50/12	33 (P) 29 (M) 0 (H)	6 (9.7)
243 (84.3)	179(≤400) 109 (>400)	18 (0) 212 (A) 19 (B) 39 (C)	87 (30.2)	5.5 ± 3.3	253/35	155 (P) 132 (M) 1 (H)	22 (7.6)
120 (83.3)	86 (≤400) 58 (>400)	10 (0) 107 (A) 8 (B) 19 (C)	44 (30.5)	5.6 ± 3.8	128/14	76 (P) 66 (M) 2 (H)	11 (7.6)
340 (60.0)	297 (<20) 270 (≥20)	151 (0) 416 (A)	157 (27.7)	2.7 (2.0–4.0)^b^	545/22	193 (P) 353 (M) 21 (H)	22 (3.9)
333 (58.7)	296 (<20) 271 (≥20)	142 (0) 425 (A)	148 (26.1)	2.8 (2.0–4.1)^b^	540/27	194 (P) 360 (M) 13 (H)	22 (3.9)
37 (29.36)	109 (10.3–1,210)^a^	NA	NA	4.4 (2.6–8.5)^a^	NA	NA	NA
59 (55.14)	97.5 (7.8–1,210)^a^	NA	NA	3.8 (2.8–9.7)^a^	NA	NA	NA

Note: PO cohort, in this cohort, patients received entecavir or tenofovir post-operation; PPO cohort, in this cohort, patients received entecavir or tenofovir pre-operation and post-operation; NOS, Newcastle–Ottawa scale; R, retrospective study; RCT, randomized controlled trial; PSM, propensity score matching; PS overlap weighting, propensity-score overlap weighting; IPTW, inverse probability of treatment weighting; ETV, entecavir; TDF, tenofovir; HBV, hepatitis B virus; NA, not available; HBeAg, hepatitis B e antigen; AFP, alpha-fetoprotein; BCLC, Barcelona clinical liver cancer; MVI, microvascular invasion; P, poorly differentiated; M, moderately differentiated; H, highly differentiated.

^a^ data are presented as the median and range.

^b^ data are presented as the median and inter-quartile range.

### Quality assessment

Supplementary Tables S2, S3 list the quality assessment details of the included studies. Of the nine non-RCT studies, two scored 7 points, two scored 8 points, and five scored 9 points. Thus, the two studies with 7 points were deemed as moderate-quality, and the remaining seven were classified as high-quality. For the RCT, the blind method implementation was not elucidated in the article, with all other domains showing low risk.

### Outcomes

**FIGURE 2:**
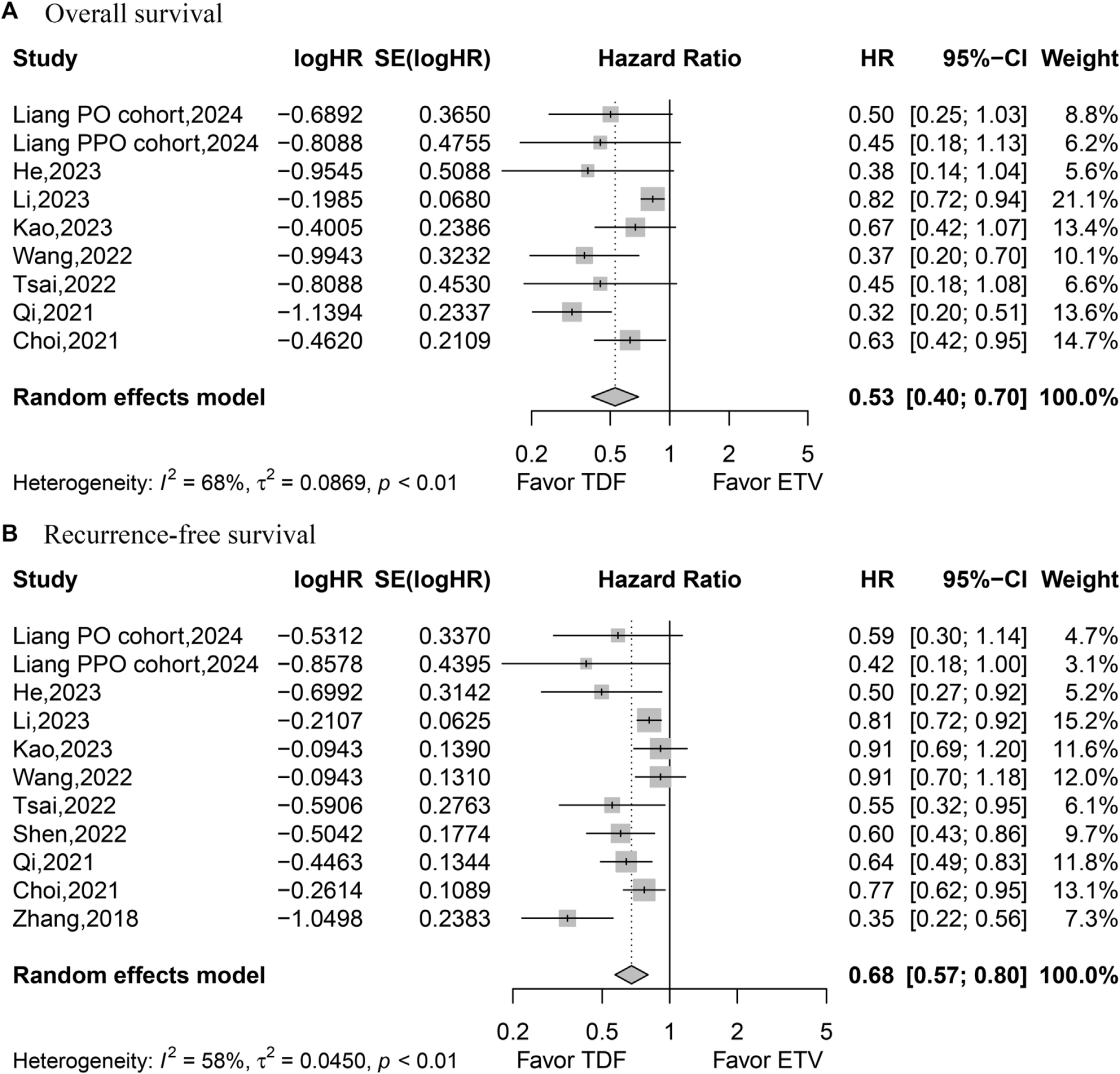
Forest plot for overall survival **(A)** and recurrence-free survival **(B)**.

HR values for OS were reported in eight studies comprising nine datasets and analyzed based on the random-effects model. The aggregated data indicated that patients treated with TDF achieved superior OS (HR, 0.53; 95% CI, 0.40–0.70; *p* < 0.0001) (Figure 2). Ten studies with 11 datasets documented the HR values for RFS, which was analyzed with the random-effects model. The combined data suggested that patients treated with TDF achieved better RFS (HR, 0.68; 95% CI, 0.57–0.80; *p* < 0.0001) (Figure 2).

**FIGURE 3:**
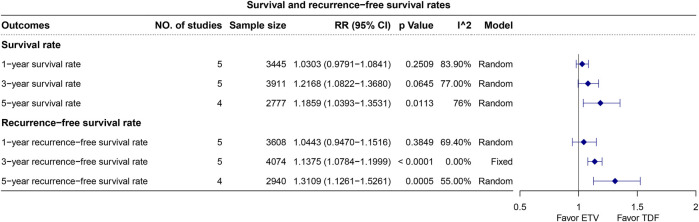
Plot for the overall survival and recurrence-free survival rates.

The 1-, 3-, and 5-year OS rates were reported in five, five, and four studies, respectively, and analyzed using the random-effects model due to the observed heterogeneity. The pooled results revealed a higher 5-year OS rate with TDF (5-year RR, 1.19; 95% CI, 1.04–1.35; *p* = 0.0113) but similar 1- and 3-year OS rates (Figure 3). Similarly, 1-, 3-, and 5-year RFS rates were documented in five, five, and four studies, respectively, and analyzed using the random-effects model, with the exception of the 3-year RFS rate, which presented low heterogeneity. The synthesized data revealed higher 3- and 5-year RFS rates with TDF (3-year RR, 1.14; 95% CI, 1.08–1.20; *p* < 0.0001; 5-year RR, 1.31; 95% CI, 1.13–1.53; *p* = 0.0005), with similar 1-year RFS rates (Figure 3).

**FIGURE 4:**
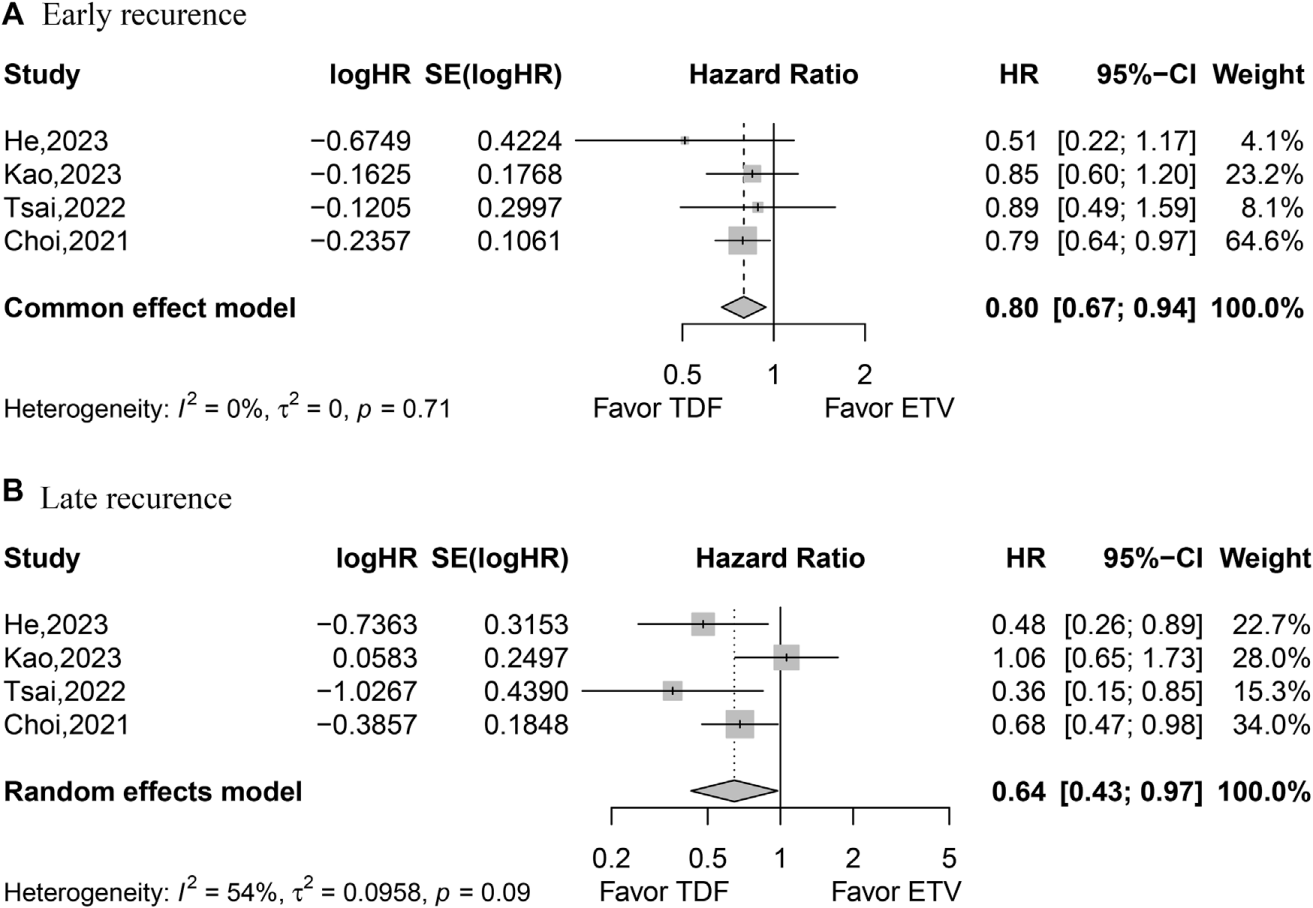
Forest plot for early recurrence **(A)** and late recurrence **(B)**.

The studies with four datasets that reported HR values for early recurrence were analyzed using the fixed-effects model. The pooled data indicated that patients treated with TDF achieved better early recurrence outcomes (HR, 0.80; 95% CI, 0.67–0.94; *p* < 0.0077) (Figure 4). Furthermore, four studies with four datasets reporting HR values for late recurrence were analyzed with the random-effects model. The combined data suggested that patients treated with TDF achieved better late recurrence (HR, 0.64; 95% CI, 0.43–0.97; *p* = 0.0368) (Figure 4).

### Meta-regression, subgroup analyses, and sensitivity analyses

**FIGURE 5:**
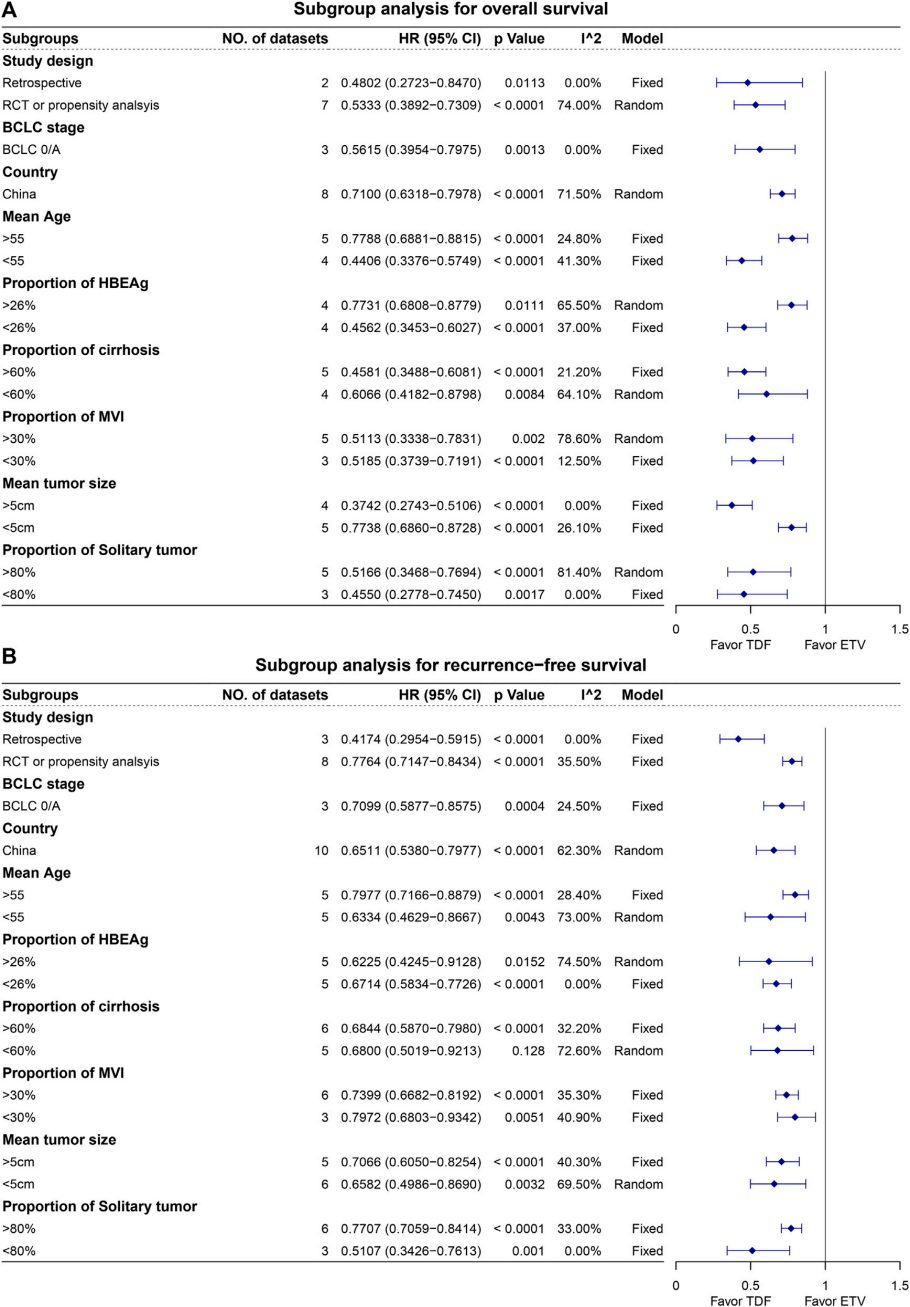
Subgroup analysis for overall survival **(A)** and recurrence-free survival **(B)**.

Meta-regression was performed only for RFS due to the smaller number of OS datasets (<10). The results revealed the sample size and retrospective study design as the heterogeneity sources (Supplementary Figure S1). Subgroup analyses were conducted for retrospective studies, propensity analysis studies combined with RCT, studies that only included BCLC early-stage HCC, studies conducted in China, and available patient characteristics. Figure 5 shows that patients receiving TDF achieved better OS and RFS in subgroup analyses. In the sensitivity analysis of OS, the overall heterogeneity decreased after the removal of the study conducted by Li, indicating that this study is one of the sources of heterogeneity but does not affect the results of the meta-analysis. This study was a propensity score matching (PSM) study with high quality and the largest sample size (Supplementary Figure S2). The sensitivity analysis for RFS indicated that the result was stable (Supplementary Figure S2B). Additionally, the sensitivity analyses indicated that the early recurrence and late recurrence results were less stable (Supplementary Figures S2C, D). The studies by Kao et al. and Choi et al. would affect the results of early recurrence and late recurrence.

### Publication bias

Funnel plots with the Egger test revealed publication bias in OS and RFS but not in early recurrence and late recurrence (Supplementary Figure S3). Contour-enhanced funnel plots for OS and RFS indicated filled studies in the white area (*p* < 0.05), suggesting that publication bias affected the meta-analysis results (Supplementary Figures S4A, B). After filling potential unpublished studies, the meta-analysis showed superior RFS for patients receiving TDF compared to ETV, while OS remained similar between TDF and ETV recipients (Supplementary Figure S5).

## Discussion

In our meta-analysis, HBV-related HCC patients on TDF exhibited better OS, RFS, early recurrence, and late recurrence. Previous meta-analyses that mainly compared TDF and ETV for HBV-related HCC prognosis (Giri et al., 2023; Liu et al., 2023; Kong et al., 2024) included patients receiving various treatments, such as liver transplantation, radiofrequency ablation, and liver resection, inevitably introducing bias. We focused solely on HCC patients undergoing liver resection to minimize bias from different treatments. Additionally, we comprehensively analyzed the result reliability. The meta-regression analysis revealed the heterogeneity sources, urging for larger, higher-quality studies. The subgroup analyses by study design and tumor stage supported our findings. Moreover, publication bias was identified. Addressing publication bias by filling the potentially unpublished studies revealed the consistently better efficacy of TDF on RFS over ETV, questioning its superiority on OS. This increases our confidence in the hypothesis that TDF can lead to better outcomes for HCC patients than ETV. Additionally, we advocate for publishing articles with negative or conflicting conclusions. Based on the meta-analysis, we believe that TDF is superior to ETV in improving RFS. The current research suggested that the improvement in RFS with TDF is due to its superior HBV-DNA suppression and anti-inflammatory effects compared to ETV (12). Therefore, TDF is more suitable as an antiviral medication for postoperative patients with higher viral loads.

Our study is subject to some constrains. First, the majority of the incorporated studies were non-RCTs. Although they demonstrated high quality, the inherent bias cannot be fully addressed by propensity analysis. Second, all studies were performed in Asia, potentially restricting the generalizability of our findings to other populations. Third, heterogeneity was present. Although we conducted a meta-regression analysis to explore its source, the subgroup analysis supported the reliability of our results. Fourth, publication bias was identified. However, using the trim-and-fill method, we observed the consistent efficacy of TDF over ETV in terms of RFS. Lastly, some studies had relatively small sample sizes, potentially impacting the robustness of our findings.

## Conclusion

Our meta-analysis demonstrated the superiority of TDF over ETV in RFS for HBV-related HCC patients. These findings carry significant implications for postoperative antiviral therapy selection. However, further extensive, high-quality RCTs are necessary to bolster evidence and draw more conclusive recommendations.

## Data Availability

The original contributions presented in the study are included in the article/Supplementary Material; further inquiries can be directed to the corresponding author.
