# Supplementary material for: A systematic review and meta-analysis comparing the impact of tenofovir and entecavir on the prognosis of hepatitis B virus-related hepatocellular carcinoma patients undergoing liver resection
Source: Front Pharmacol. 2024 Jul 29;15:1443551. doi: 10.3389/fphar.2024.1443551 (PMC11317427; doi:10.3389/fphar.2024.1443551)

Table S1

| Databases | Search strategy |
| --- | --- |
| Pubmed 29 | #1 Hepatocellular carcinoma[MeSH Terms] OR hepatocellular carcinoma OR HCC  #2 tenofovir OR TDF  #3 entecavir OR ETV  #4 hepatectomy[MeSH Terms] OR liver resection OR hepatic resection OR hepatectomy  #5 #1 AND #2 AND #3 AND #4 |
| Embase 82 | #1 'liver cell carcinoma'/exp OR 'hepatocellular carcinoma':ti,ab,kw OR hcc:ti,ab,kw  #2 'tenofovir'/exp OR tenofovir:ti,ab,kw OR tdf:ti,ab,kw  #3 'entecavir'/exp OR entecavir:ti,ab,kw OR etv:ti,ab,kw  #4 'hepatectomy'/exp OR 'liver resection':ti,ab,kw OR 'hepatic resection':ti,ab,kw OR hepatectomy:ti,ab,kw  #5 #1 AND #2 AND #3 AND #4 |
| Web of Science 24 | #1 **(TS=(hepatocellular carcinoma)) OR TS=(hcc)**  **#2 (TS=(tenofovir)) OR TS=(tdf)**  **#3 (TS=(entecavir)) OR TS=(etv)**  **#4 ((TS=(liver resection)) OR TS=(hepatic resection)) OR TS=(hepatectomy)**  #5 #1 AND #2 AND #3 AND #4 |
| Cochrane Library 12 | #1 MeSH descriptor: [Carcinoma, Hepatocellular] explode all trees  #2 (hepatocellular carcinoma):ti,ab,kw OR (hcc):ti,ab,kw  #3 #1 OR #2  #4 MeSH descriptor: [Tenoforvir]explode all trees  #5 (tenoforvir):ti,ab,kw OR (tdf):ti,ab,kw  #6 #4 OR #5  #7 MeSH descriptor: [Entecavir]explode all trees  #8 (entecavir):ti,ab,kw OR (etv):ti,ab,kw  #9 #7 OR#8  #10 MeSH descriptor: [Hepatectomy]explode all trees  #11 (liver reseciton):ti,ab,kw OR (hepatic resection):ti,ab,kw OR (hepatectomy):ti,ab,kw  #12 #10 OR #11  #13 #3 AND #6 AND #9 AND #12 |

Table S2 Risk assessment of RCT

| Study | Random sequence generation | Allocation concealment | Blinding of outcome assessment | Blinding of participants and personnel | Incomplete outcome data | Selective reporting | Other bias |
| --- | --- | --- | --- | --- | --- | --- | --- |
| He 2023 | Low Risk | Low Risk | Unclear | Unclear | Low Risk | Low Risk | Low Risk |
|  |  |  |  |  |  |  |  |

Table S3 NOS score of non-RCT studies

| Study | Selection | | | | Comparability | Outcome | | | Total score |
| --- | --- | --- | --- | --- | --- | --- | --- | --- | --- |
|  | Representativeness  of the  exposed cohort | Selection of  the non- exposed cohort | Ascertainment  of exposure | Demonstration  of outcome |  | Assessment of outcome | Follow-up was long enough | Adequacy  of follow up |  |
| Liang 2024 PO cohort | * | * | * | * | * | * |  | * | 7 |
| Liang 2024 PPO cohort | * | * | * | * | * | * |  | * | 7 |
| Li 2023 | * | * | * | * | ** | * | * | * | 9 |
| Kao 2023 | * | * | * | * | ** | * | * | * | 9 |
| Wang 2022 | * | * | * | * | ** | * |  | * | 8 |
| Tsai 2022 | * | * | * | * | * | * | * | * | 8 |
| Shen 2022 | * | * | * | * | ** | * | * | * | 9 |
| Qi 2021 | * | * | * | * | ** | * | * | * | 9 |
| Choi 2021 | * | * | * | * | * | * | * | * | 9 |
| Zhang 2018 | * | * | * | * | * | * |  | * | 7 |
|  |  |  |  |  |  |  |  |  |  |

Figure S1. The plot of meta-regression for recurrence-free survival. A. Sample size; B. Study design.

A.


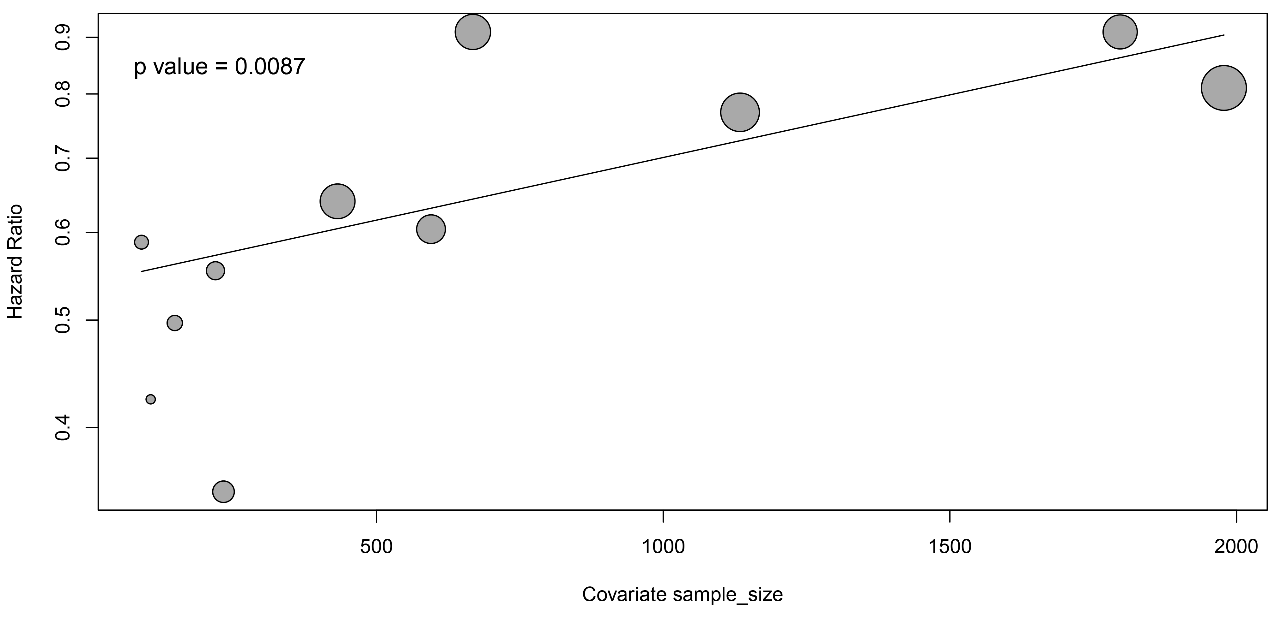


B.


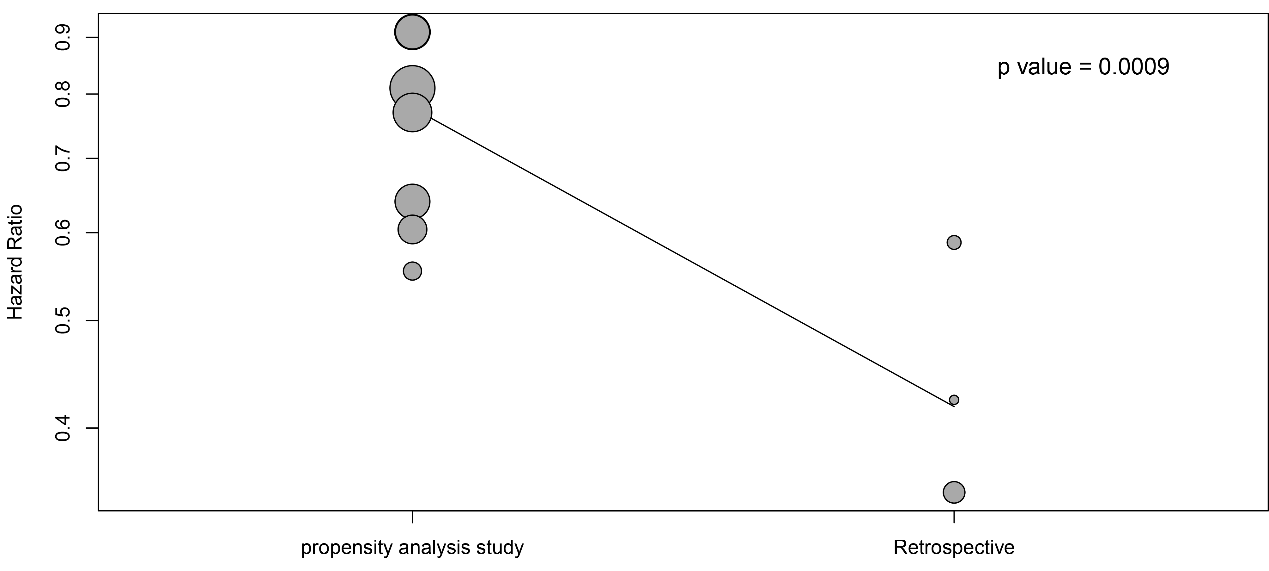


Figure S2. The plot of sensitivity analyses for overall survival, recurrence-free survival, early recurrence, and late recurrence. A. overall survival; B. recurrence-free survival; C, early recurrence; D, late recurrence.

A.
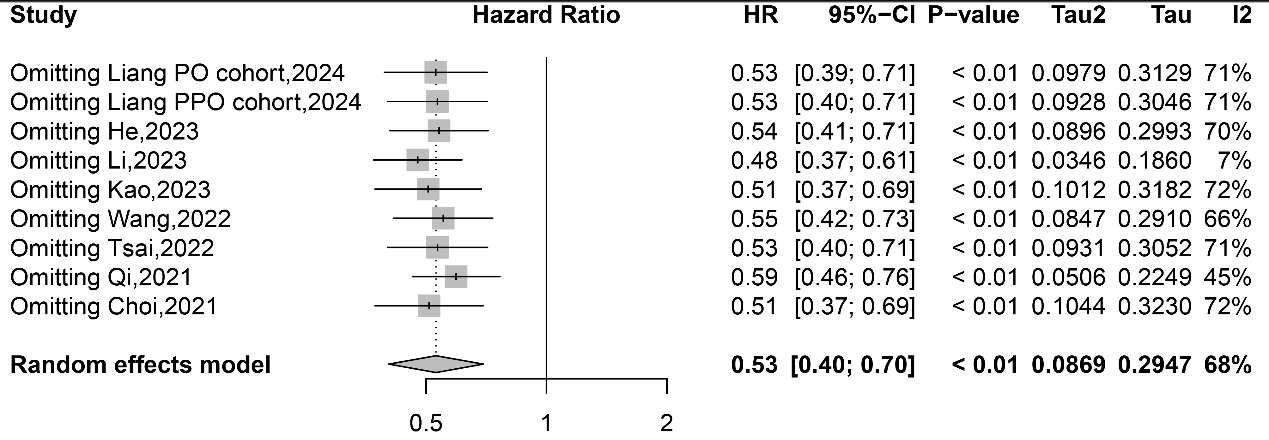


B.
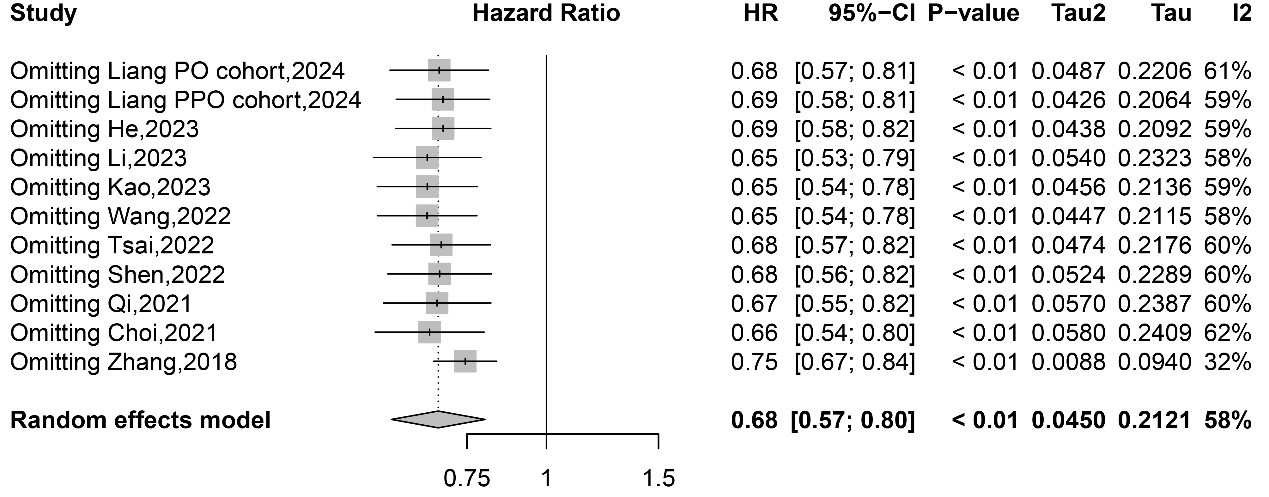


C.
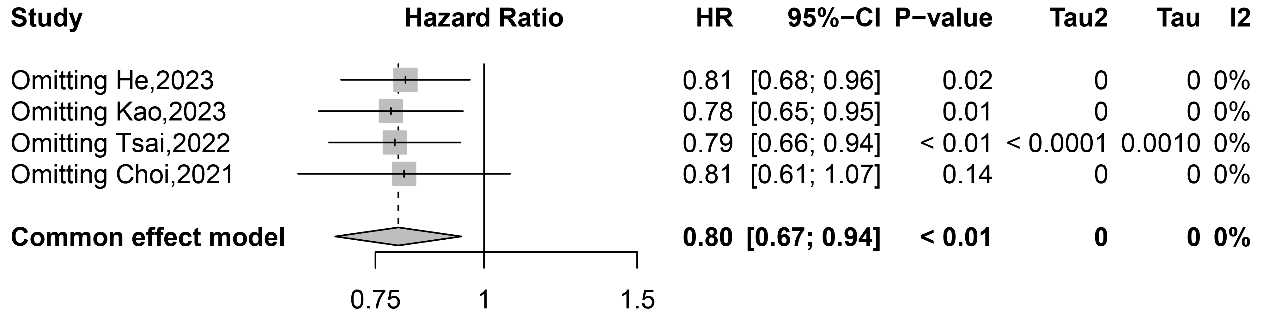


D.
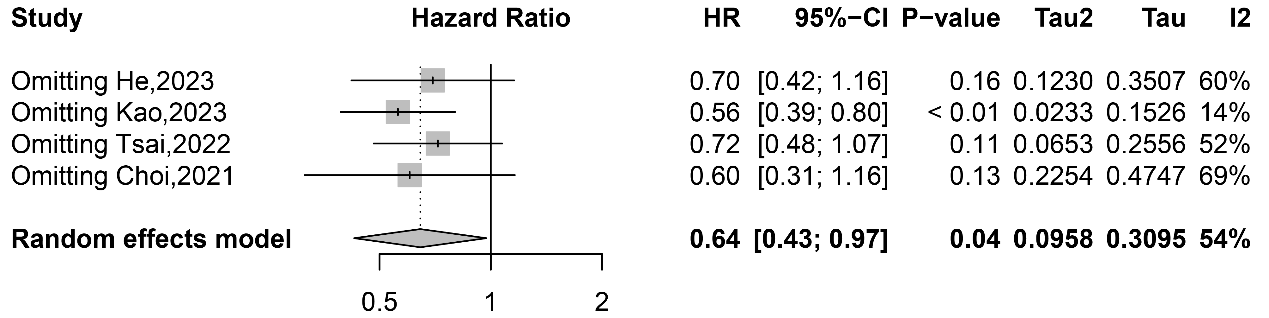


Figure S3. The funnel plot for overall survival, recurrence-free survival, early recurrence, and late recurrence. A. overall survival; B. recurrence-free survival; C, early recurrence; D, late recurrence.

A.
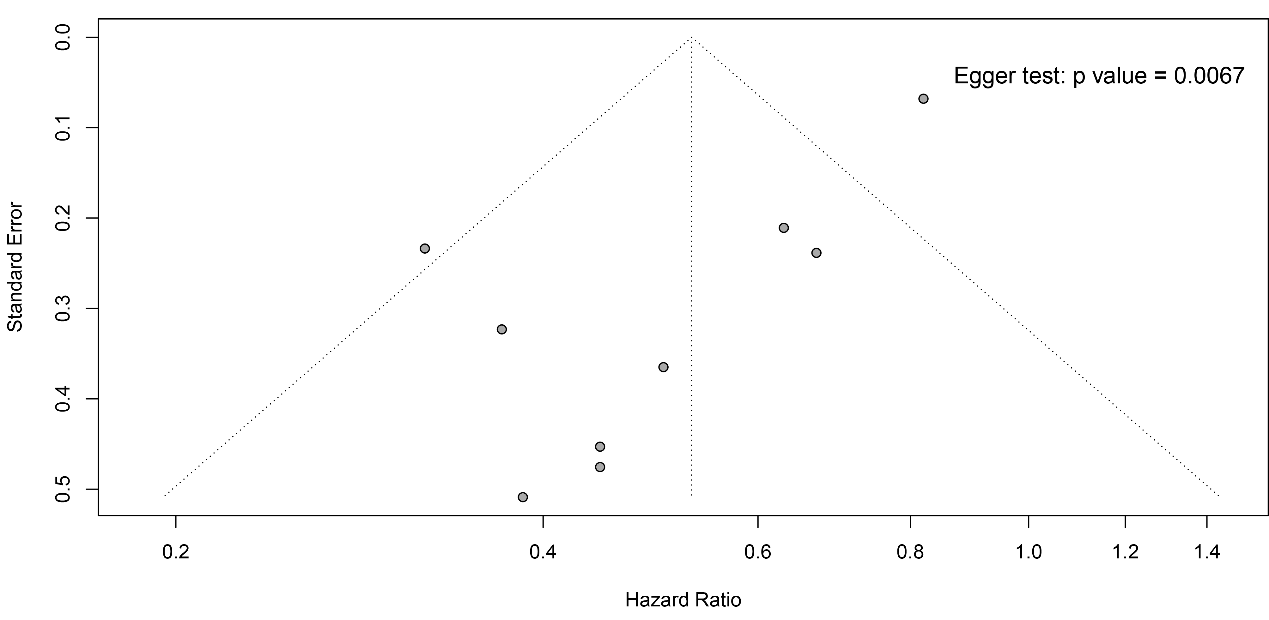


B.
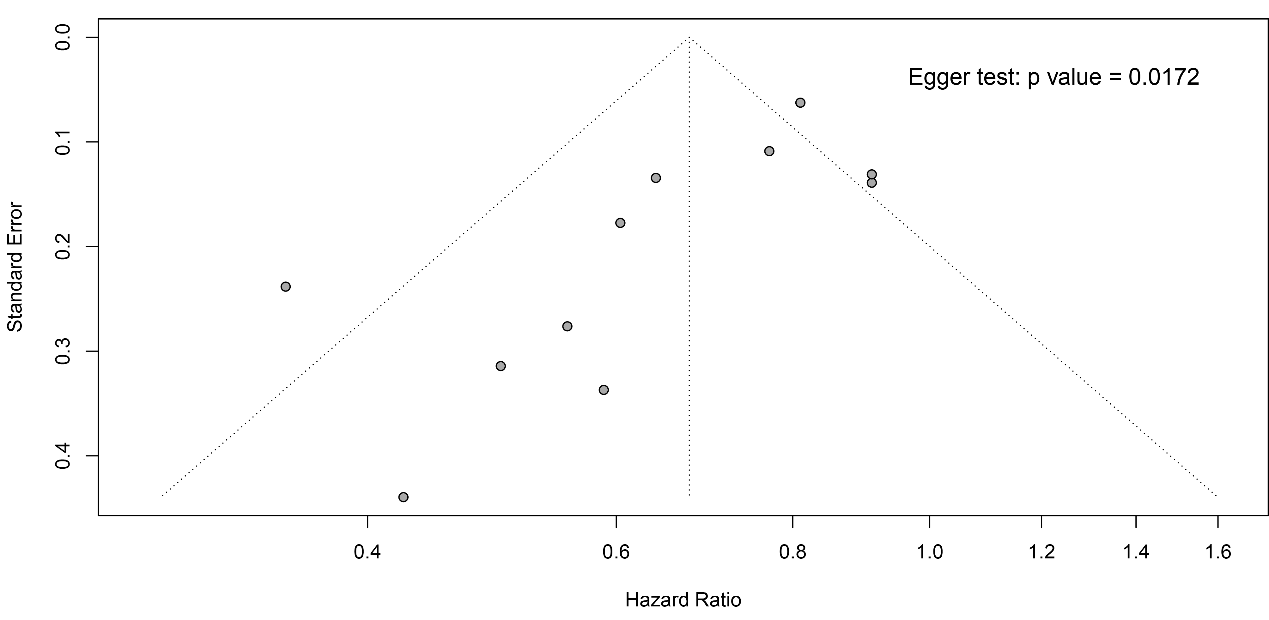


C.
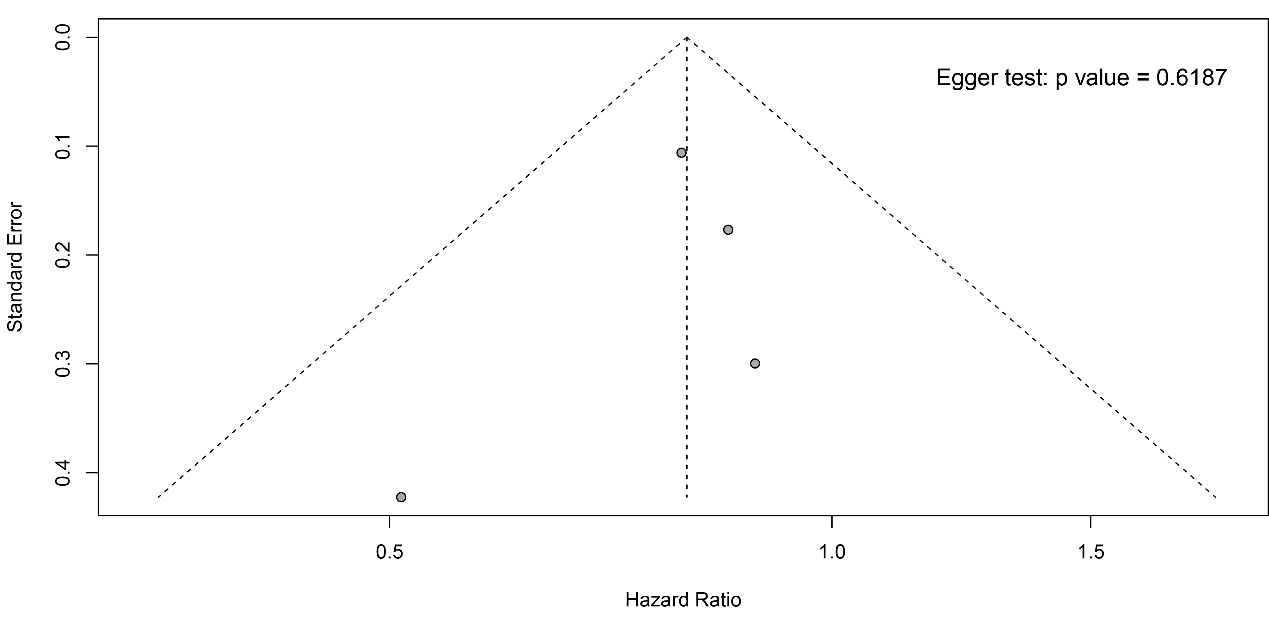


D.
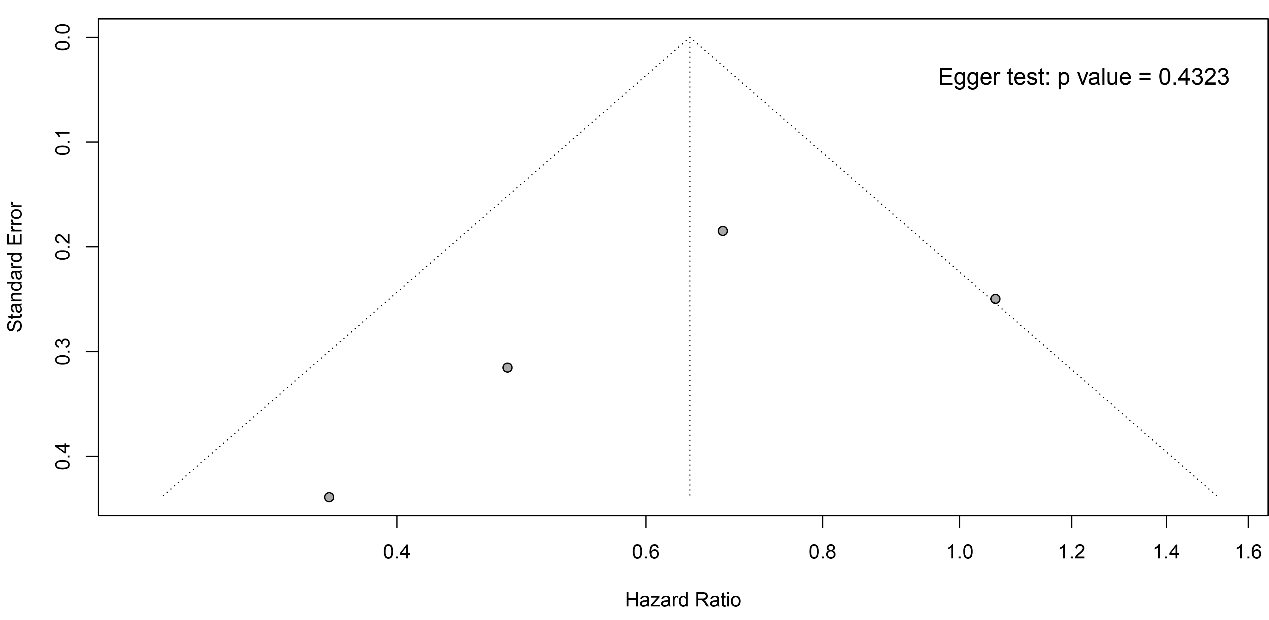


Figure S4. The Contour-enhanced funnel plot for overall survival and recurrence-free survival. A. overall survival; B. recurrence-free survival.

A.
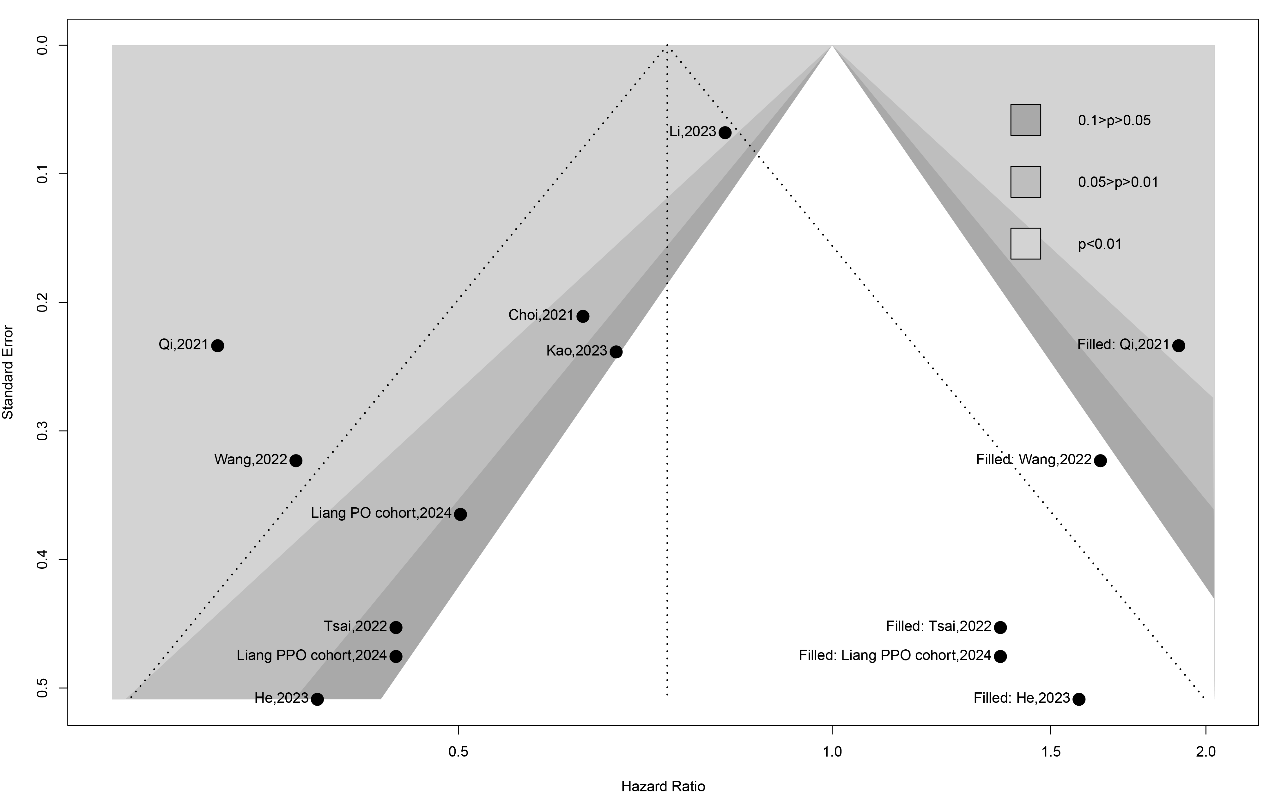


B.
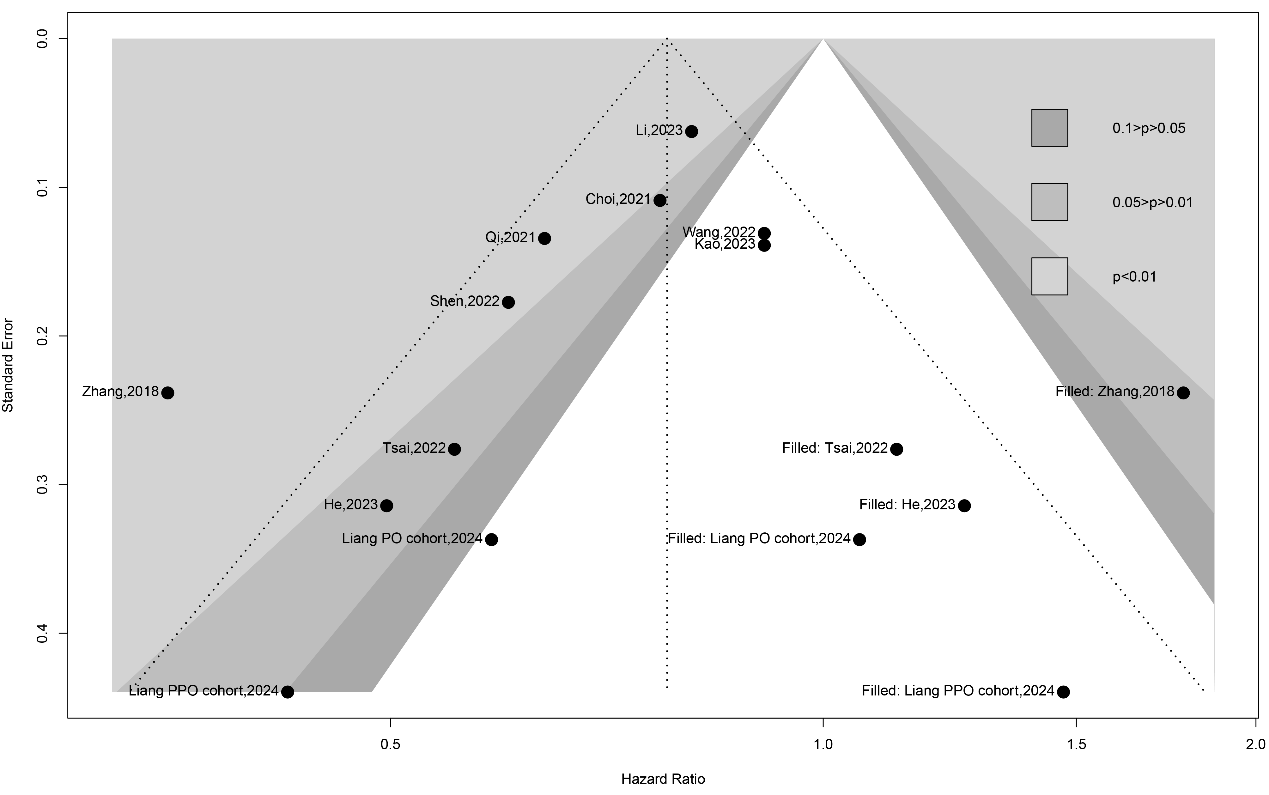


Figure S5. The forest plot for overall survival and recurrence-free survival after filling potential unpublished articles. A. overall survival; B. recurrence-free survival.


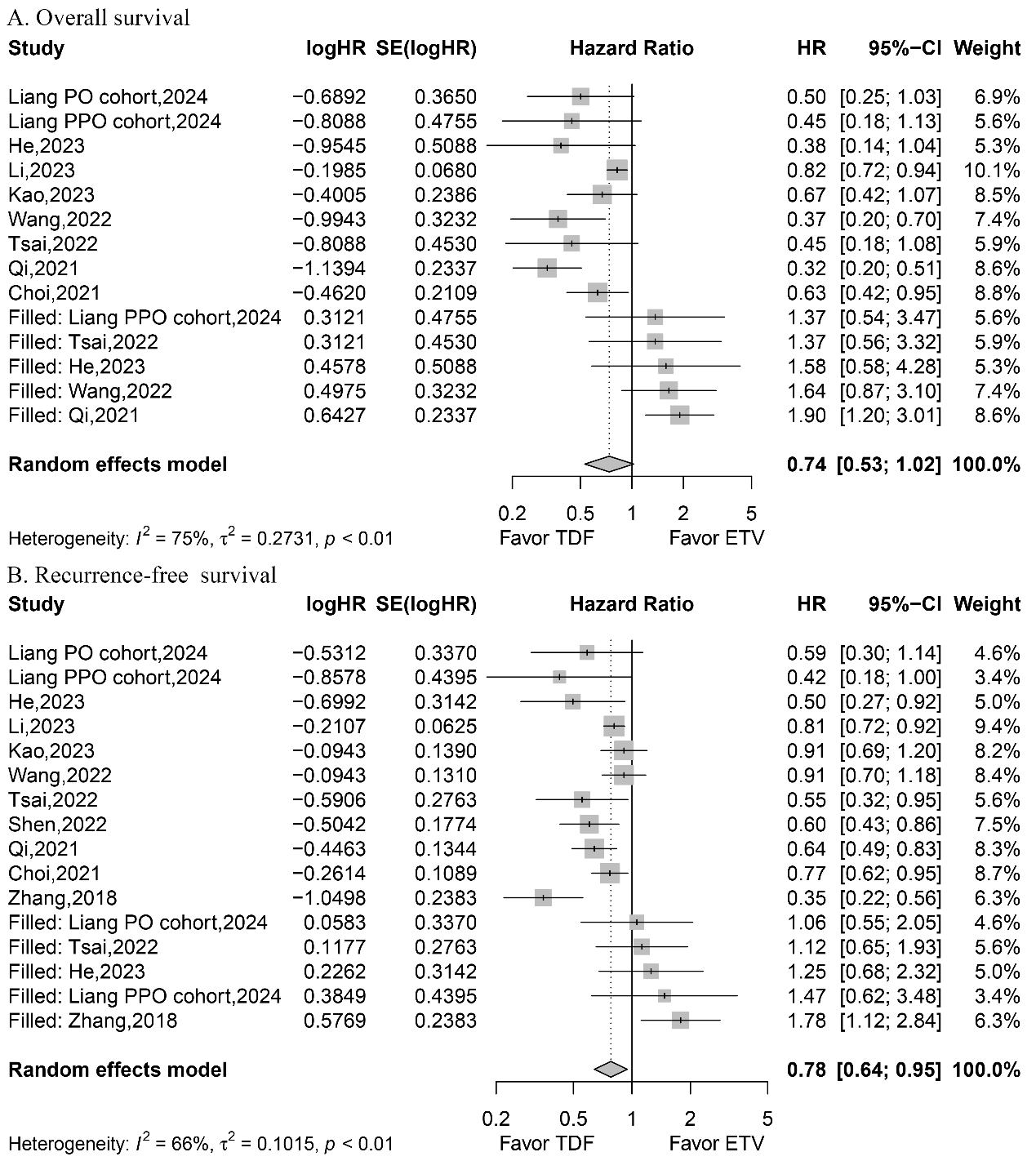

Supplement: Supplementary file 1 [file DataSheet1.docx]
